# Consequences of Adolescent Exposure to the Cannabinoid Receptor Agonist WIN55,212-2 on Working Memory in Female Rats

**DOI:** 10.3389/fnbeh.2017.00137

**Published:** 2017-07-21

**Authors:** Erin K. Kirschmann, Daniel M. McCalley, Caitlyn M. Edwards, Mary M. Torregrossa

**Affiliations:** ^1^Department of Psychiatry, Translational Neuroscience Program, University of Pittsburgh Pittsburgh, PA, United States; ^2^Center for Neuroscience, University of Pittsburgh Pittsburgh, PA, United States

**Keywords:** cannabis, WIN55, 212-2, adolescence, female, working memory, self-administration

## Abstract

Marijuana is a prevalent illicit substance used by adolescents, and several studies have indicated that adolescent use can lead to long-term cognitive deficits including problems with attention and memory. However, preclinical animal studies that observe cognitive deficits after cannabinoid exposure during adolescence utilize experimenter administration of doses of cannabinoids that may exceed what an organism would choose to take, suggesting that contingency and dose are critical factors that need to be addressed in translational models of consequences of cannabinoid exposure. Indeed, we recently developed an adolescent cannabinoid self-administration paradigm in male rats, and found that prior adolescent self-administration of the cannabinoid receptor agonist WIN55,212-2 (WIN) resulted in improved working memory performance in adulthood. In addition, the doses self-administered were not as high as those that are found to produce memory deficits. However, given known sex differences in both drug self-administration and learning and memory processes, it is possible that cannabinoid self-administration could have different cognitive consequences in females. Therefore, we aimed to explore the effects of self-administered vs. experimenter-administered WIN in adolescent female rats on adult cognitive function. Female rats were trained to self-administer WIN daily throughout adolescence (postnatal day 34–59). A control group self-administered vehicle solution. The acute effects of adolescent WIN self-administration on memory were determined using a short-term spatial memory test 24 h after final SA session; and the long-term effects on cognitive performance were assessed during protracted abstinence in adulthood using a delayed-match-to-sample working memory task. In a separate experiment, females were given daily intraperitoneal (IP) injections of a low or high dose of WIN, corresponding to self-administered and typical experimenter-administered doses, respectively, or its vehicle during adolescence and working memory was assessed under drug-free conditions in adulthood. While self-administration of WIN in adolescence had no significant effects on short-term spatial memory or adult working memory, experimenter administration of WIN resulted in improved adult working memory performance that was more pronounced in the low dose group. Thus, low-dose adolescent WIN exposure, whether self-administered or experimenter-administered, results in either improvements or no change in adult working memory performance in female rats, similar to previous results found in males.

## Introduction

Legal policy in the U.S. regarding both medicinal and recreational use of marijuana has become increasingly relaxed over the last decade. Considering that the prevalence of marijuana use among adolescents is between 35 and 40 percent (Moss et al., 2014; Salas-Wright et al., 2016), it is crucial to understand the long-term effects of marijuana exposure during adolescence in order to properly inform any potential policy changes. Adolescence is a period of substantial neuronal development. Of particular importance, the development of both the prefrontal cortex, a brain region required for higher-order cognitive processing, and the endocannabinoid system, responsible for modulating various physiological functions as well as the binding of exogenous cannabinoids, occur simultaneously (Spear, 2000; Schneider, 2008). The primary psychoactive components of marijuana are cannabinoids, particularly Δ^9^-tetrahydrocannabinol (THC), that activate cannabinoid receptors CB1 and CB2. Therefore, considering the concurrent changes in neuronal and cannabinoid system development, there is potential for marijuana use to cause cannabinoid-induced interference in the carefully orchestrated development of the adolescent brain, possibly resulting in long-term effects on cognition.

Several studies have been conducted in order to assess the long-term effects of cannabinoid use during adolescence; however, the findings have been mixed. Some clinical work has linked adolescent THC use to reduced IQ, increased risk for psychosis, and impaired working memory (Meier et al., 2012; Becker et al., 2014; Gage et al., 2016; Marconi et al., 2016). In contrast, other clinical studies have failed to find differences in performance between cannabis users and controls on cognitive tasks (Jager et al., 2006; Buchy et al., 2015; Mokrysz et al., 2016). In preclinical animal studies, the most common application of cannabinoids has been via experimenter administration, which does not model the volitional control over intake and choice over dose of intake observed in human populations, bringing to question the translational value of these studies. Nevertheless, these studies have generally found a relationship between chronic cannabinoid exposure and cognitive deficits, especially in working memory, object recognition and short-term spatial memory capacity (Hampson and Deadwyler, 2000; O’Shea et al., 2004, 2006; Schneider and Koch, 2007; Abush and Akirav, 2012; Renard et al., 2016).

We recently developed a model of adolescent intravenous (IV) cannabinoid self-administration, which more closely models the voluntary nature of drug use in humans (Kirschmann et al., 2017). We trained rats to self-administer the synthetic cannabinoid receptor agonist WIN55,212-2 (WIN), a full agonist of CB1 and CB2 cannabinoid receptors that is more potent than THC, because previous studies have found that rats will readily self-administer this compound, whereas rats did not readily self-administer THC, which is a partial agonist (Takahashi and Singer, 1979; Fattore et al., 2001; Deiana et al., 2007; Pertwee, 2010; Lefever et al., 2014). Although WIN’s effects may be more directly comparable to the frequently abused synthetic cannabinoids such as K2 and spice, it provides us with the opportunity to investigate the effects of self-directed cannabinoid receptor activation on behavior. Interestingly, adolescent self-administration of WIN did not lead to acute memory deficits or long-term effects on working memory performance using a delayed-match-to-sample task. In fact, we found that rats that self-administered WIN had better working memory performance in adulthood (after several weeks of abstinence) than control animals that responded for sucrose pellets. However, the doses of WIN that rats were willing to self-administer were much lower than doses used in previous studies identifying memory impairments after experimenter-administration. Thus, it is possible that cannabinoid-induced memory deficits are likely to only be found in very heavy users, and/or are not likely to be observed after long periods of abstinence. Another potential difference between findings in rodents and humans is that almost all previous preclinical work, including our own study, was conducted in male subjects. Given that there are known sex differences in both drug self-administration and learning and memory processes, it is possible that adolescent cannabinoid exposure could produce different results in males and females. Therefore, the current study aimed to explore the long-term cognitive effects of self-administered versus experimenter-administered cannabinoids in adolescent female rats.

## Materials and Methods

### Animals

A total of 42 female, Sprague–Dawley (Harlan, Frederick, MD, USA) rats were delivered on postnatal day (PND) 22 and were housed in a climate-controlled room on a 12-h dark/light cycle (lights on at 4:30 am) throughout the duration of the experiment. All behavioral experiments were conducted during the light phase. Rats were pair-housed unless otherwise indicated, and were food restricted to about 85%–90% of normal, free-feeding weight for the duration of all behavioral experiments. Food portions were adjusted daily throughout adolescence with females receiving a range of 13–15 g/day. Food restriction was necessary to maintain responding in behavioral tasks. All procedures were performed in accordance with the recommendations of the National Institutes of Health *Guide for the Care and Use of Laboratory Animals*. The protocol was approved by the University of Pittsburgh’s Institutional Animal Care and Use Committee.

### Drugs

The synthetic cannabinoid receptor agonist WIN55,212-2 mesylate (WIN; NIMH Chemical Synthesis and Drug Supply Program; Cayman Chemical Company, Ann Arbor, MI, USA), was dissolved in sterile 0.9% saline with a drop of Tween 80. Fresh stock solutions were made every 2–3 days, and were diluted with saline daily to a working concentration for IV administration of 0.0125 mg/kg/infusion. A vehicle solution of 0.9% saline and a drop of Tween 80 was created daily and was diluted with saline for IV administration and intraperitoneal (IP) administration. Fresh working solutions of 1.2 mg/mL and 0.2 mg/mL were prepared daily for IP administration.

### Surgical Procedures

In animals responding for IV WIN/vehicle, surgery was performed to implant indwelling jugular catheters on PND 27–28. Rats were anesthetized with ketamine (90 mg/kg) and xylazine (5 mg/kg) and given 5 mg/kg of the analgesic Rimadyl. Catheters were constructed with silastic tubing (11 cm; Braintree Scientific, Braintree, MA, USA) attached to a bent steel guide cannula (22 gauge; Plastics One Inc., Roanoke, VA, USA), surrounded by Loctite medical epoxy (Grainger, Pittsburgh, PA, USA) and attached to a 2 cm square piece of mesh (Bard Mesh; Davol Inc., Cranston, RI, USA). Catheters were implanted in the right jugular vein and fed subcutaneously to the back, where they exited through a small incision between the shoulder blades, and were flushed daily with 0.1 ml of Gentamicin in heparinized saline. A recovery period of 7 days was allowed before initiation of behavioral testing.

### Self-Administration

**Figure 1:**
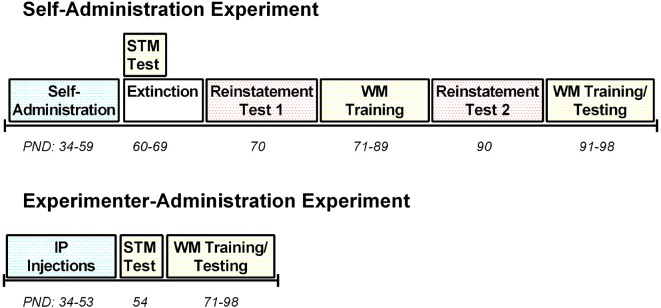
Timeline of treatment and behavioral testing for both the self-administration and experimenter-administration experiments. PND, postnatal day; STM, short-term memory; WM, working memory; IP, intraperitoneal.

Between PNDs 34–59, rats (*n* = 10) were trained to self-administer the synthetic cannabinoid receptor agonist WIN in standard operant conditioning chambers (Med Associates, St. Albans, VT, USA; see Figure 1 for an experimental timeline). The chambers contained two retractable levers, a house light, two stimulus lights, a tone generator, a food magazine and a fan for background noise. A press on the active lever resulted in a 0.0125 mg/kg/infusion delivery of WIN at a rate of 0.03 ml/s paired with a 10 s tone and light stimulus in combination with a 10 s timeout, during which the house light was extinguished. Infusion times were adjusted daily based on individual rats’ weights to maintain the 0.0125 mg/kg/infusion dose. A press on the inactive lever yielded no programmed consequences. Rats were trained on a 1-h fixed-ratio 1 (FR1) schedule of reinforcement during the first 4 days of self-administration, and were then switched to 2-h sessions (FR1) for the remaining 18 days of self-administration. Control rats (*n* = 8) responded for IV infusions of a vehicle solution using identical training procedures as for WIN.

### Extinction and Reinstatement Testing

Following the last day of self-administration training, rats underwent instrumental extinction (1-h sessions), where lever presses produced no cue presentations or IV infusions. Rats underwent extinction for 8–9 days and until the rat had fewer than 20 lever presses. The day following the last day of extinction, and again 20 days later, rats were tested for cue-induced reinstatement of WIN seeking in 30 min sessions. During these sessions, lever presses resulted in a 10 s presentation of the audiovisual cue on an FR1 schedule. No infusions were given during reinstatement.

### Experimenter-Administration

In a separate experiment, female rats (*n* = 24) received IP injections daily throughout adolescence (PND 34–53) between 2:00–3:00 pm during the light phase (Figure 1). Rats received either vehicle (*n* = 8), a low dose of WIN, 0.2 mg/kg, which corresponds to the average total daily dose that males received during self-administration (Kirschmann et al., 2017; *n* = 8), or a high dose of WIN, 1.2 mg/kg, which corresponds to the dose commonly given in previous experiments that identified deficits in short-term memory (Schneider and Koch, 2003; O’Shea et al., 2006; *n* = 8). The total number of days of exposure to WIN was almost the same between the experimenter-administration and self-administration groups, though the age at which exposure ended varied by 6 days. The self-administration group did not have sessions on ~3 days during the study due to experimental conflicts, and rats that self-administered tended to get fewer infusions, and thus a lower dose of WIN, on the initial day of self-administration. Thus, the low dose IP group and the self-administration group are not perfectly equated, but received similar amounts of WIN overall.

### Short-Term Spatial Memory Task

All rats were allowed to habituate to an empty, open field chamber (43 cm × 43 cm; Med Associates) under dim light for a period of 5 min on three occasions. Within 24 h of the last day of WIN exposure (PND 60 for self-administration (SA) rats, PND 54 for IP rats), all rats underwent short-term spatial memory testing in the same open field chamber. Two objects were placed evenly from opposing corners in the chamber, and rats were allowed to explore the objects for a period of 5 min. Objects were similar in material (glass/ceramic) and dimension (11 cm × 11 cm). Rats were returned to their home cages, and one of the objects was moved to a new spatial location. Following a 35-min delay, rats were again allowed to explore the objects in the chamber. Typically, rats prefer novelty; hence a rat with intact spatial memory will spend more time examining the object in the novel location, compared to the time spent examining the object in the familiar location. All rats performed this task and the chamber was cleaned with 70% ethanol between trials. Data were collected and analyzed offline using AnyMaze software (Stoelting, Wood Dale, IL, USA).

### Delayed-Match-to-Sample Working Memory Task

Following 10–11 days of abstinence (which allowed the rats to age into adulthood), all rats began training in a delayed-match-to-sample working memory task. Training was performed during 1-h sessions in chambers (Med Associates) equipped with five nose poke apertures and a food dispenser. Initially, all five apertures were illuminated and rats received a sucrose pellet (45 mg, Bioserv, Flemington, NJ, USA) reward upon response on any of these five holes on an FR1 schedule. Next, a single aperture was illuminated and only a response in that hole resulted in reward. On the following days of training, a response on the single, illuminated “sample” aperture resulted in the immediate illumination of the “sample” and two additional “choice” apertures. The sample aperture for each trial was selected at random using Med Associates random number generator software code. The additional two apertures illuminated during the choice phase were always the two closest apertures to the sample, resulting in the two flanking apertures for the middle three apertures, and the next two innermost apertures for outer apertures. Following illumination of the choice aperture, a second response on the “sample” resulted in sucrose reward (FR1). Responses in any of the other apertures resulted in a 2 s timeout where all lights were extinguished. Once animals performed this task reliably with accuracy greater than 75% correct, delays were introduced between the “sample” phase and the “choice” phase. Rats performed blocks of trials in which seven delays (0.5–6 s) were presented in random order; each of the seven delays occurred before a new block of trials began. Once rats reached training criterion (≥80% correct 0.5 s delay), the range was increased (0.5–12 s; 0.5–24 s; See Tables 1, 2 for summaries of SA and IP groups’ training data). Working memory was assessed most critically on the first day in which the animals were presented with delays between 0.5 s and 24 s, ranging from age PND 93 to 98.

**Table 1:** Training data for self-administration study.

	Days to meet criteria^a^		Reinforcers earned^b,c,d^	Accuracy across delays^e^
Phase 1	WIN	1.0 (±0)	60.1 (±4.3)	N/A
	VEH	1.0 (±0)	62.6 (±6.5)	
Phase 2	WIN	4.0 (±0.3)	59.1 (±6.0)	N/A
	VEH	4.1 (±0.1)	63.8 (±9.8)	
Phase 3	WIN	4.0 (±0.4)	58.2 (±2.3)	N/A
	VEH	3.9 (±0.3)	61.3 (±6.7)	
Phase 4	WIN	5.6 (±0.6)	49.9 (±2.3)	*F*_(1,16)_ = 0.51, *p* = 0.49
	VEH	5.6 (±0.5)	54.1 (±3.4)	
Phase 5	WIN	6.0 (±0.4)	47.2 (±2.4)	*F*_(1,16)_ = 0.29, *p* = 0.60
	VEH	6.0 (±0.2)	56.6 (±3.3)*	

^a^Number of days WIN SA or VEH SA rats remained in current phase of training before advancing to next phase. Reinforcers earned: ^b^Single day of phase 1; ^c^Average over first 4 days in phases 2–3; ^d^Average over first 6 days in phases 4–5. ^e^Accuracy across delays on first day of training in phases 4–5. *p < 0.05, WIN vs. VEH.

**Table 2:** Training data for experimenter-administration study.

	Days to meet criteria^a^		Reinforcers earned^b,c,d^	Accuracy across delays^e^
Phase 1	Low	1.0 (±0)	23.9 (±2.8)	N/A
	High	1.0 (±0)	27.3 (±2.8)	
	Veh	1.0 (±0)	26.9 (±3.1)	
Phase 2	Low	7.0 (±0.6)	24.4 (±6.3)	N/A
	High	7.1 (±0.4)	18.4 (±4.4)*	
	Veh	6.0 (±0.5)	41.0 (±6.4)	
Phase 3	Low	3.9 (±0.7)	63.6 (±10.6)	N/A
	High	2.9 (±0.1)	84.0 (±7.6)	
	Veh	3.0 (±0.2)	95.3 (±23.7)	
Phase 4	Low	3.4 (±0.3)	57.5 (±5.3)	*F*_(2,21)_ = 0.47, *p* = 0.63
	High	4.0 (±0.3)	48.2 (±6.1)	
	Veh	4.0 (±0.3)	54.7 (±7.7)	
Phase 5	Low	5.6 (±0.3)	50.6 (±3.6)	*F*_(2,21)_ = 0.78, *p* = 0.47
	High	5.8 (±0.4)	45.8 (±5.8)	
	Veh	6.6 (±0.5)	52.0 (±6.0)	

^a^Number of days Low WIN (0.2 mg/kg) IP, High WIN (1.2 mg/kg) IP or vehicle IP rats remained in current phase of training before advancing to next phase. Reinforcers earned: ^b^Single day of phase 1; ^c^Average over first 6 days in phases 2 and 5; ^d^Average over first 3 days in phases 3–4. ^e^Accuracy across delays on first day of training in phases 4–5. *p < 0.05, High WIN vs. VEH.

### Estrous Cycle Monitoring

Vaginal lavage samples were taken by gently pipetting 150 μl of saline into the vagina to flush the vaginal canal. The saline was pipetted onto a clean microscope slide and covered with a coverslip. Cell morphology was examined under a compound microscope at a magnification of 100–200× to determine estrous cycle phase using standard procedures (Goldman et al., 2007). In order to allow for uninterrupted pubertal development, vaginal lavage sampling began on PND 46 (day 13 of the self-administration period) and was performed daily until the final day of self-administration. Samples were also taken on days of significant testing, specifically on the first day of each new delay range in working memory. Females were split into two groups of either high estradiol (proestrus or transitioning in or out of proestrus; PRO), or low estradiol (estrus, metestrus, or diestrus; EMD) and analyzed accordingly.

### Statistical Analyses

Data were analyzed using Prism 6.0 Software (GraphPad Inc., LaJolla, CA, USA) and the SPSS software package version 21.0 (SPSS, Chicago, IL, USA). Self-administration and extinction data were analyzed using repeated measures (rm) ANOVA with a within-subjects factor of day of self-administration and between-subjects factor of group (WIN vs. VEH SA). Additionally, separate rmANOVA analyses of VEH and WIN SA groups were conducted with a within-subjects factor of day of self-administration and between-subjects factor of response type (active lever vs. infusions vs. inactive lever). Reinstatement data were analyzed by comparing within-subjects lever pressing on the last day of extinction to pressing across days of abstinence, comparing responses on the active and inactive levers by rmANOVA. Short-term spatial memory and working memory were also analyzed by rmANOVA, comparing test phase or delay length within-subjects and adolescent treatment type (WIN vs. VEH) between subjects. Fisher’s least significant difference (LSD) test was used for *post hoc* analyses following identification of significant effects. Two-sided *t*-tests were used to determine if high or low estradiol phases of the estrous cycle [proestrus (PRO) vs. a combination of estrus, metestrus, and diestrus (EMD)] affected self-administration behaviors during the last 5 days of training.

## Results

### Adolescent Self-Administration, Extinction and Cue-Induced Reinstatement

**Figure 2:**
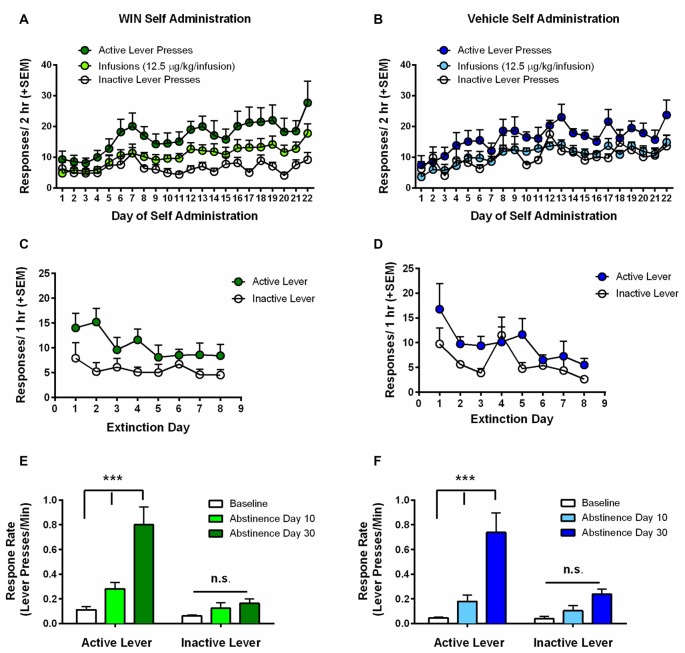
Adolescent self-administration behaviors. **(A)** Rats self-administering WIN throughout adolescence showed clear discrimination between active and inactive lever responses over days, and rats earned significantly more infusions relative to inactive lever presses. **(B)** Rats self-administering vehicle showed a smaller, but significant differentiation between active and inactive lever responses, but no difference between inactive lever presses and infusions earned. **(C,D)** Significant extinction of the lever press response occurred in both the WIN **(C)** and VEH **(D)** groups. **(E)** Rats that self-administered WIN exhibited significant cue-induced reinstatement of lever pressing over response rates on the last day of extinction (i.e., baseline) on both 10 and 30 days of abstinence. Reinstatement on day 30 was also significantly greater than that observed on day 10. **(F)** Rats that self-administered VEH did not exhibit a significant cue-induced reinstatement of lever pressing on day 10, but responding on day 30 was significantly greater than baseline and day 10 response rates. ^***^p < 0.001.

Analysis of infusions earned during self-administration by rmANOVA with group (WIN vs. VEH) as the between subjects factor showed that infusion number increased over the course of self-administration, regardless of whether responding was for WIN or for vehicle solution (VEH; main effect of SA day, *F*_(21,336)_ = 5.41, *p* < 0.001; no group × day interaction *F*_(21,336)_ = 0.51, *p* = 0.97). We also conducted separate rmANOVAs for WIN and VEH groups individually in order to compare the pattern of active vs. inactive lever pressing across days under each self-administration condition. Adolescent female rats that self-administered WIN reliably discriminated between the active and inactive levers (Figure 2A). Analysis by rmANOVA found a significant main effect of response type across days (*F*_(2,27)_ = 14.83, *p* < 0.001), and Fisher’s LSD test indicated significant difference between inactive lever presses and both active lever presses (*p* < 0.001) and infusions earned (*p* = 0.041). Females responding for VEH did not significantly differ from WIN treated females in the number of active or inactive lever presses overall, and exhibited a significant main effect of response type (*F*_(2,21)_ = 15.30, *p* < 0.001). Further analysis indicated that only active and inactive lever presses significantly differed (*p* < 0.001), while there was no difference between infusions earned and inactive lever presses (*p* = 0.65; Figure 2B). Therefore, inactive pressing for VEH did not separate from infusions earned to the same degree as in the WIN group, though there were no overall between-drug differences. We hypothesize that the high degree of VEH responding was likely driven by presentation of the audiovisual cue. Importantly, the equatable levels of SA performance during adolescence in the VEH group serves as a better behavioral control for our later assessment of cognitive consequences, since both groups had similar experience with the cues. Females self-administered an average dose of 0.15–0.16 mg/kg WIN over the last 5 days of self-administration, similar to what we have previously observed in males, where the average daily dose was 0.22 mg/kg (Kirschmann et al., 2017).

Next, we examined extinction of the lever press response, where active lever presses produced neither the IV infusion, nor the audiovisual cue. Analysis of active lever responding by rmANOVA with day as within-subjects factor and group (WIN vs. VEH) as between subjects factor showed no effect of SA condition on extinction behavior (main effect of day (*F*_(7,112)_ = 4.02, *p* = 0.001), no group × day interaction (*F*_(7,112)_ = 1.08, *p* = 0.384)). We confirmed in separate rmANOVAs for WIN and VEH groups that both WIN and VEH self-administration groups exhibited a significant decrease in active lever responding over days (main effects of day (*F*_(7,126)_ = 2.57, *p* = 0.017) and (*F*_(7,98)_ = 3.90, *p* < 0.001), respectively), indicating acquisition of extinction (Figures 2C,D). However, the WIN group did not extinguish to as low of a degree of responding as the VEH group, exhibiting a significant difference between active and inactive lever presses across days of training (*F*_(1,18)_ = 4.70, *p* = 0.044), while the VEH group showed no significant differences between active and inactive lever responses over time (*F*_(1,14)_ = 2.80, *p* = 0.12).

Following extinction training, rats were tested for cue-induced reinstatement in two 30-min sessions where active lever presses resulted in presentation of the audiovisual cue. The first test occurred following the last day of extinction training, on day 9–10 of abstinence depending on when the rat met the extinction criterion. The second test occurred 20 days later, corresponding to day 29–30 of abstinence. Due to the difference in session length between extinction training and reinstatement tests, response rates were calculated (lever presses/minute) and compared between the last day of extinction (baseline) and the two reinstatement tests. Analysis by rmANOVA with group (WIN vs. VEH) as between-subjects factor identified a main effect of test phase (*F*_(2,32)_ = 44.52, *p* < 0.001), but no effect of group (*F*_(1,16)_ = 0.616, *p* = 0.44) or group × test phase interaction (*F*_(2,32)_ = 0.04, *p* = 0.96). Analysis of WIN and VEH SA groups separately by rmANOVA revealed that the WIN self-administration group exhibited significant differences based on test phase (*F*_(2,18)_ = 26.09, *p* < 0.001), and lever type (*F*_(1,9)_ = 17.42, *p* = 0.002), and a significant interaction between the two (*F*_(2,18)_ = 23.60, *p* < 0.001). *Post hoc* analysis indicated that there were no significant differences in inactive lever response rates across test days, but that there was a significant difference between baseline responding (i.e., on the last day of extinction) and response rates on day 10 (*p* = 0.018) and day 30 (*p* < 0.001) of abstinence, indicating significant cue-induced reinstatement of WIN seeking on both test days. Furthermore, there was also a significant increase in responding from day 10 to day 30 of abstinence (*p* < 0.001), indicating a significant incubation of craving effect (Figure 2E). Similar effects were observed in the VEH self-administration group, with significant effects of test day (*F*_(2,14)_ = 28.02, *p* < 0.001), lever type (*F*_(1,7)_ = 9.48, *p* = 0.018) and their interaction (*F*_(2,14)_ = 7.28, *p* = 0.007). However, on day 10 of “abstinence”, there was no significant difference in response rate from the extinction baseline (*p* = 0.20). Nevertheless, on day 30 of “abstinence”, response rates were significantly increased over both baseline (*p* < 0.001) and day 10 levels (*p* < 0.001). Therefore, while there was not a significant cue-induced reinstatement effect on day 10, there was still an increase in cue-motivated responding on day 30, despite the cue never being paired with a drug (Figure 2F). These data suggest that cues can be strong motivators of behavior (at least in adolescent females), independent of conditioning to a reinforcer.

### Estrous Cycle Phase Does Not Impact Self-Administration

**Figure 3:**
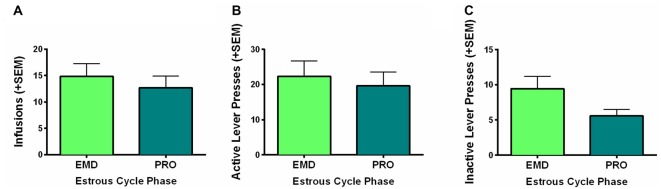
Role of estrous cycle phase on WIN self-administration. Behaviors during the high estradiol phase of proestrus (PRO) were compared to behavior during the low estradiol phases of estrus, metestrus and diestrus (EMD). No significant differences were identified for **(A)** infusions earned, **(B)** active lever presses, or **(C)** inactive lever presses.

The present study was conducted in freely cycling females, thus making it difficult to explicitly assess the relationship between circulating hormones and motivation to self-administer WIN. However, we did monitor estrous cycle phase via observation of vaginal cytology during the last 9 days of self-administration and averaged the infusions earned, active and inactive lever presses of rats during the last 5 days of self-administration on days that they were in a high estradiol phase (PRO) and a low estradiol phase (EMD). Using two-sample *t*-tests (PRO vs. EMD), we found no significant differences based on estrous cycle in infusions (*t*_(16)_ = 0.639, *p* = 0.53) or active lever presses (*t*_(16)_ = 0.445, *p* = 0.66; Figures 3A,B). Inactive lever presses did appear to be reduced during proestrus, but this only reached a trend level of significance (*t*_(16)_ = 1.783, *p* = 0.09; Figure 3C).

### Adolescent Cannabinoid Exposure Does Not Acutely Affect Short-Term Spatial Memory

**Figure 4:**
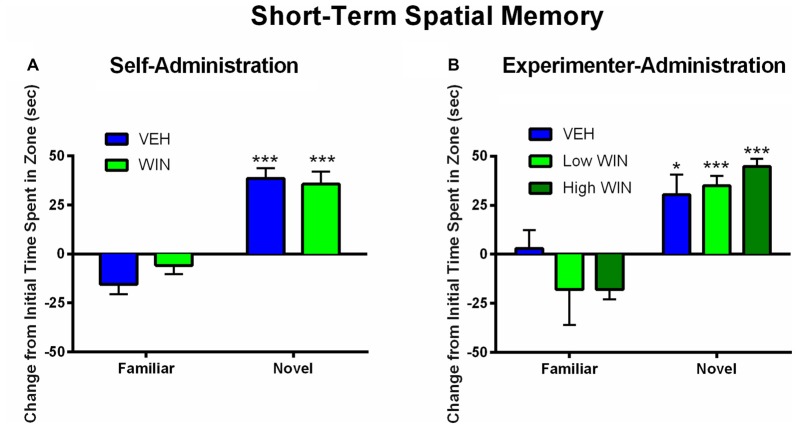
Effect of adolescent WIN exposure on short-term spatial memory. **(A)** Self-administration of WIN did not result in any acute deficits in short-term spatial memory in an object location task in female rats tested 24 h after the last self-administration day. All rats showed a significant discrimination between the familiar and novel spatial locations, ^***^p < 0.001. **(B)** Experimenter-administration of either a low or high dose of WIN did not significantly alter short-term spatial memory 24 h after the last injection day. All groups exhibited significant discrimination between familiar and novel locations, indicating intact memory, *p ≤ 0.05, ^***^p < 0.001.

We next tested whether or not females would exhibit acute deficits in short-term spatial memory 24 h following the last self-administration session. Since previous research has indicated that adolescent experimenter administration of WIN, at doses substantially higher than those obtained during self-administration, can produce short-term memory deficits in males (Kirschmann et al., 2017), a separate cohort of rats was exposed to chronic WIN in adolescence using an experimenter-administration procedure. We compared a dose of WIN comparable to that obtained during self-administration (0.2 mg/kg/day) to the commonly used “high” dose of WIN (1.2 mg/kg/day). Neither self-administration, nor experimenter-administration of WIN at either dose, produced any short-term memory deficits in females. Both WIN and VEH self-administration groups exhibited equivalent discrimination of novel from familiar spatial locations (main effect of location (*F*_(1,13)_ = 68.09, *p* < 0.001), but no effect of treatment (*F*_(1,13)_ = 0.08, *p* = 0.78; Figure 4A). Similar results were observed under experimenter-administration conditions, with a main effect of location (*F*_(1,20)_ = 43.19, *p* < 0.001), but no effect of treatment (*F*_(2,20)_ = 0.29, *p* = 0.75; Figure 4B).

### Working Memory Performance

**Figure 5:**
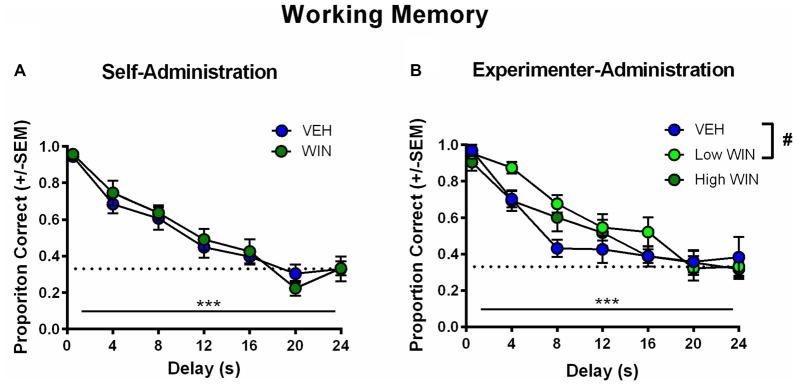
Effect of adolescent WIN exposure on adult working memory. **(A)** Adult working memory performance in rats that self-administered WIN or VEH in adolescence. All working memory training and testing was conducted under drug-free conditions. As delay increased, performance decreased, ^***^p < 0.001. Adolescent WIN self-administration had no significant effect on working memory performance. **(B)** Adult working memory performance in rats that were exposed to WIN in adolescence via experimenter-administration (IP injection) of a low dose (0.2 mg/kg/day) or a high dose (1.2 mg/kg/day), or its vehicle. In all rats, as delay increased, performance decreased, ^***^p < 0.001. The low dose of WIN produced a strong trend toward improved working memory performance relative to vehicle, and separate analyses of delays from 4 s to 16 s, indicated a significant improvement in performance, ^#^*p* = 0.05.

All rats began training on the delayed-match-to-sample working memory task 10–11 days after the last drug exposure day, which followed extinction and initial reinstatement testing in the self-administration group. All groups received between 22 days and 25 days of training prior to final working memory assessments. We observed no differences between the self-administration groups across different phases of training (Table 1). Analysis of working memory performance once rats reached the final test phase, where the delays between sample and choice phases are longest, revealed no significant effects of treatment (*F*_(1,17)_ = 0.01, *p* = 0.91). However, we did observe the expected effect of delay, with performance accuracy significantly decreasing as the delay length was increased (*F*_(6,102)_ = 44.99, *p* < 0.001; Figure 5A). Thus, female rats that self-administered WIN during adolescence showed neither an improvement nor a detriment in working memory performance in adulthood under drug-free conditions.

Conversely, analysis of working memory performance in rats that received experimenter-administered WIN in adolescence indicated that exposure to the low, but not the high dose, produced working memory improvements in adulthood (see Table 2 for training data). Analysis by rmANOVA identified the expected decrease in performance with increasing delay length (*F*_(6,126)_ = 40.29, *p* < 0.001), and a strong trend toward a treatment effect when all three treatment groups were included in the analysis (*F*_(2,21)_ = 3.19, *p* = 0.06). Due to the fact that the potential treatment effect appeared to be driven by low dose WIN exposure and differences are not expected at very short and very long delays, a separate two-way rmANOVA comparing the vehicle and low dose groups across 4–16 s delays was conducted, which indicated a significant improvement in working memory performance after adolescent exposure to low dose WIN (*F*_(1,14)_ = 10.57, *p* = 0.006; Figure 5B). Similar analysis of the high WIN dose group did not reveal a significant treatment effect on performance (*F*_(1,14)_ = 2.34, *p* = 0.15).

## Discussion

The aims of the present study were to determine the long-term effects of self-administered cannabinoids in adolescence on adult cognitive function, and to explore the abuse liability of adolescent-onset cannabinoid use, in females. Additionally, we aimed to explore whether similar cognitive consequences arose from different routes of cannabinoid administration during adolescence. Similar to our previous findings in males, there were no detrimental effects of adolescent WIN self-administration on adult cognitive performance in females. Additionally, experimenter-administration of a low dose of WIN in adolescent females resulted in improved performance in a delayed-match-to-sample working memory task in adulthood. Together, our data provide further support towards the notion that cannabinoid exposure under self-administration conditions, or at behaviorally-relevant doses, is less detrimental than originally suspected.

### Analysis of Female Cannabinoid Self-Administration

The National Institutes of Health now requires the consideration of sex as a relevant biological variable in all new applications for funding. To our knowledge, our current study in females, in conjunction with our previously published findings in males (Kirschmann et al., 2017), is one of the first analyses of males and females in adolescent cannabinoid self-administration (but see work in adults by Fattore et al., 2007, 2010). We show here that adolescent female rats will self-administer the synthetic cannabinoid receptor agonist WIN55,212-2, and will attain a daily dose similar to, though slightly less than, the daily dose that what male rats attained in our prior study (~0.15 mg/kg here in females vs. 0.22 mg/kg in males; Kirschmann et al., 2017). This is in contrast to rodent findings for other drugs of abuse such as cocaine, ethanol and opiates, where females take substantially higher amounts compared to males (e.g., Becker et al., 2007; Anker and Carroll, 2010; Becker and Koob, 2016; Bertholomey et al., 2016). However, our finding that females maintained a slightly lower daily dose of a cannabinoid receptor agonist than males is in line with human findings in which women report greater sensitivity to the subjective effects of inhaled or orally-administered cannabis (Cooper and Haney, 2014; Fogel et al., 2017). There were no significant differences in self-administration behaviors during the high estradiol phase (proestrus) compared to the low estradiol phases (estrus, metestrus and diestrus), indicating a lack of a role of estrous cycle in cannabinoid self-administration in adolescent females, at least to the degree to which we could assess estrous cycle in this study. In contrast, Fattore et al. (2007) found that ovariectomized adult females self-administered significantly less WIN compared to intact controls; however different strains and ages of rats were used in that study, and ovariectomy clamps estradiol at much lower levels than what occurs naturally during all phases of the estrous cycle, which could explain the difference in findings.

Female rats that self-administered WIN showed significant cue-induced lever pressing after a short abstinence period (10 days); and they exhibited even greater cue-induced lever pressing after an extended abstinence period (30 days), indicating an “incubation of craving” effect for a self-administered cannabinoid, similar to our previous findings in male rats (Kirschmann et al., 2017). However, female rats that self-administered the vehicle solution also exhibited increased cue-induced lever pressing after an extended period of “abstinence” (or lack of exposure to vehicle or cues). Few groups have ever examined incubation of craving in a vehicle group, particularly in females. The closest work may be that of Lee et al. (2013), and that of Werner et al. (2015), in which male rats trained to self-administer saline showed a lack of reinstatement for visual stimuli previously paired with infusion delivery; although in one case, if all reinstatement test days were collapsed, saline-trained animals did show a memory for the saline cue (Werner et al., 2015). Experiments with rodents responding for natural rewards (e.g., sucrose or food pellets) also have demonstrated reinstatement and incubation of craving for the cues previously paired with the natural rewards (e.g., Grimm et al., 2011; Darling et al., 2016; Dingess et al., 2017). Additionally, our paradigm utilized an audiovisual cue, and compound stimuli have themselves been shown to serve as conditioned reinforcers (Fuchs et al., 1998; See et al., 1999; Caggiula et al., 2002). Finally, adolescents have been shown to learn more about cues than adults (e.g., Meyer and Bucci, 2016). Thus, the increased cue-induced reinstatement after 30 days of abstinence in female rats that had previously self-administered the vehicle solution in adolescence is likely a reflection of compound cues serving as strong motivators of behavior in adolescent females, and makes it difficult to interpret if the incubation of craving observed in the WIN group is related specifically to the potential reinforcing property of WIN or to the compound cues. Future studies could examine if self-administration of other unit doses of WIN would produce greater self-administration and reinstatement in females, though the high level of responding in the vehicle group is still an important factor to consider when interpreting reinstatement and incubation of craving results across drug classes.

### Cannabinoid Exposure in Adolescence Differentially Impacts Cognition

Similar to what we have shown in males, WIN self-administration did not cause acute deficits in short-term spatial memory in females. Contrary to findings in males (Abush and Akirav, 2012; Kirschmann et al., 2017), experimenter-administration of a high dose of WIN (1.2 mg/kg) in adolescence did not result in acute deficits in short-term spatial memory in females. This could reflect the differential sensitivity of females to cannabinoids; potentially, exposure to a higher or lower dose of WIN than was used in this study could have resulted in acute short-term memory deficits in females. We then examined the long-term effect of adolescent WIN exposure and we found a significant improvement in working memory in females that received the low dose (0.2 mg/kg) during adolescence. In contrast to what we previously reported in males, adolescent self-administration of WIN in females did not significantly impact adult working memory performance. It is not clear if this is due to sex differences in the impact of self-administered vs. experimenter-administered drugs, or if the lack of effect in the self-administration group is due to the fact that the females self-administered a lower dose of WIN (~0.15 mg/kg/day) than males. Thus, a very tight dose-effect relationship may exist between adolescent WIN exposure and improvements in adult working memory performance. Nevertheless, under no conditions by dose, sex, or route of administration have we observed long-lasting deficits in working memory after adolescent WIN exposure.

### Limitations and Conclusions

The present set of experiments does have some caveats. First, it is clear that adolescent females will respond for cues, regardless of the solution they receive IV. This limits our interpretation of the self-administration data on the degree to which WIN is reinforcing in and of itself. However, because adolescent females responding for IV WIN tended to show better discrimination between the active and inactive levers, there is some indication that WIN carries at least somewhat greater reinforcing value than the vehicle solution. Future work that includes a full dose-response curve and examines differences in responding with and without cues for both male and female adolescents in the same experiment would be warranted. Additionally, because our main question was focused on the long-term cognitive consequences of cannabinoid self-administration during adolescence, the fact that the vehicle group exhibited similar levels of responding during SA provides for a more closely-matched control group. All rats received equivalent exposure to the cues, and therefore the differences we observed in later working memory performance can be attributed to the WIN exposure itself. Second, because we did not include males and females in a singular experiment, definitive conclusions about sex differences in cannabinoid self-administration and effects of adolescent cannabinoid exposure on later cognitive performance cannot be made. Overall, the response rates are similar in the females of the current experiment and the males we previously tested (Kirschmann et al., 2017); a larger study that includes both sexes run simultaneously would be necessary to confirm this. Third, all of our females were food restricted for the duration of behavioral experiments. Though this was necessary to maintain motivation to complete the tasks, this could interact with our behavioral findings. In line with this, we did not conduct a full panel of cognitive assessments in our rats. Future work could aim to complete additional investigations of cognitive ability using paradigms that do not require food restriction to ensure task performance. Fourth, all of our experiments utilized the synthetic cannabinoid receptor agonist WIN55,212-2, not the main psychoactive component of marijuana, Δ^9^-THC. Although WIN does act on the same receptors as THC, its pharmacological properties may be distinct from THC, and its chemical structure may be more comparable to the frequently-abused synthetic cannabinoids K2 and spice (Pertwee, 2010). Nevertheless, there is evidence for similar effects of experimenter-administered THC or WIN on short-term memory that are blocked by nonselective cannabinoid receptor antagonists (Hampson and Deadwyler, 2000; O’Shea et al., 2004, 2006; Schneider and Koch, 2007; Abush and Akirav, 2012; Renard et al., 2016), suggesting that any effects of THC on cognition are likely to be recapitulated by WIN. Finally, we acknowledge that conclusions about self- vs. experimenter-administration are limited, in that these groups experience different daily conditions. The animals receiving daily IP injections were not exposed to any behavioral chambers prior to the start of working memory training, whereas the animals self-administering entered the behavioral chambers daily during the course of adolescence. Future work could include animals that receive IP injections but are then allowed daily exposure to the behavioral chambers, to ensure that experience is equated prior to working memory training. Despite the IP females taking a longer amount of time to reach training criteria than SA females in earlier phases of working memory training, by the time delays were introduced, all animals were performing at similar levels (see Tables 1, 2).

In conclusion, we report one of the first examinations of the effects of adolescent cannabinoid self-administration on cognitive function in females. We have found that adolescent cannabinoid exposure alone is not sufficient to produce long-lasting deficits in memory function. Our data are potentially consistent with human literature showing an increased sensitivity to cannabinoids in females, which may explain why females self-administered slightly less WIN, in contrast to other drugs of abuse. Finally, our data show some evidence for craving-like behavior after adolescent cannabinoid SA in females, suggesting the potential for abuse liability.

## Author Contributions

EKK and MMT contributed to the design of the experiments. DMM, EKK and CME were involved in data collection. EKK, DMM and MMT analyzed and interpreted the data with input from CME. EKK and DMM wrote the article with input from CME and MMT. All authors gave final approval for publication.

## Conflict of Interest Statement

The authors declare that the research was conducted in the absence of any commercial or financial relationships that could be construed as a potential conflict of interest.
